# Real-Time Vehicle Detection from UAV Aerial Images Based on Improved YOLOv5

**DOI:** 10.3390/s23125634

**Published:** 2023-06-16

**Authors:** Shuaicai Li, Xiaodong Yang, Xiaoxia Lin, Yanyi Zhang, Jiahui Wu

**Affiliations:** College of Intelligent Equipment, Shandong University of Science and Technology, Taian 271019, China; 202183230037@sdust.edu.cn (S.L.); 202283230019@sdust.edu.cn (Y.Z.); 202183230013@sdust.edu.cn (J.W.)

**Keywords:** vehicle detection, UAV imagery, YOLOv5, feature fusion network, Soft-NMS

## Abstract

Aerial vehicle detection has significant applications in aerial surveillance and traffic control. The pictures captured by the UAV are characterized by many tiny objects and vehicles obscuring each other, significantly increasing the detection challenge. In the research of detecting vehicles in aerial images, there is a widespread problem of missed and false detections. Therefore, we customize a model based on YOLOv5 to be more suitable for detecting vehicles in aerial images. Firstly, we add one additional prediction head to detect smaller-scale objects. Furthermore, to keep the original features involved in the training process of the model, we introduce a Bidirectional Feature Pyramid Network (BiFPN) to fuse the feature information from various scales. Lastly, Soft-NMS (soft non-maximum suppression) is employed as a prediction frame filtering method, alleviating the missed detection due to the close alignment of vehicles. The experimental findings on the self-made dataset in this research indicate that compared with YOLOv5s, the mAP@0.5 and mAP@0.5:0.95 of YOLOv5-VTO increase by 3.7% and 4.7%, respectively, and the two indexes of accuracy and recall are also improved.

## 1. Introduction

The usage of small, low-altitude UAVs has snowballed in recent years [1,2,3,4]. Objection detection techniques based on UAVs equipped with vision sensors have attracted much interest in areas such as unmanned vehicles and intelligent transportation systems [5,6,7,8]. UAV-based aerial vehicle detection techniques are less expensive than cameras installed at fixed locations and produce more extensive image views, greater flexibility, and broader coverage. UAVs can monitor road traffic over any range and provide critical information for subsequent intelligent traffic supervision tasks such as traffic flow calculation, unexpected accident detection, and traffic situational awareness. However, the vast percentage of vehicle targets have few feature points and small sizes [9,10], which presents a difficulty for precise and real-time vehicle detection in the UAV overhead view [11].

Existing vehicle detection approaches can be roughly divided into traditional and deep learning-based vehicle detection algorithms. Traditional vehicle detection algorithms must extract features [12,13] manually and then use SVM, AdaBoost, and other machine learning methods for classification. However, this way is time-consuming and can only extract shallow features, which has significant limitations when applied to aerial photography scenes with small targets. In recent years, with the continuous development of deep learning techniques, various artificial intelligence algorithms based on convolutional neural networks have played a great role in different fields, such as autonomous driving [14], optimization of medicine policies [15], and wildlife census [16]. Deep learning-based target detection algorithms have also been extensively applied, mainly including two-stage and single-stage algorithms. Two-stage target detection algorithms need to extract candidate regions first and then perform regression localization and classification of targets, with common examples including: Fast R-CNN [17], Faster R-CNN [18], and R-FCN [19]. Singh et al. [20] used Fast R-CNN-optimized samples to design a real-time intelligent framework that performs well on vehicle detection tasks with complex backgrounds and many small targets. Nevertheless, the model may not fit well for cases where the objective sizes vary widely. The authors of [21] conducted a study on vehicle detection based on Faster R-CNN, and the improved model reduced the latency and enhanced the detection performance for small targets. However, the model requires high computational resources in the detection process. Kong et al. [22] use a parallel RPN network combined with a density-based sample assigner to improve the detection of vehicle-dense areas in aerial images. However, the model structure is complex and requires two stages to complete the detection, which cannot meet the requirement of real-time detection. Since the two-stage detection algorithm requires the pre-generation of many pre-selected boxes, it is highly accurate but slow and cannot meet the needs of real-time detection [23]. The single-stage target detection algorithm directly transforms the localization and classification problem into a regression problem, which has an absolute speed advantage and accuracy potential compared with the two-stage one. The mainstream single-stage target detection algorithms mainly include the YOLO (You Only Look Once) series [24,25,26,27] and the SSD series [28]. Yin et al. [29] obtained outstanding detection performance for small objects by improving the efficiency of SSD in using feature information at different scales. However, the default box needs to be selected manually, which may affect the performance of the model in detecting small targets. Lin et al. [30] detect oriented vehicles in aerial images based on YOLOv4, and the improved model significantly improved the detection performance in scenarios with densely arranged vehicles and buildings. However, further improvement studies are lacking for scenes with small targets. Adel et al. [31] compared the detection performance of Faster R-CNN, YOLOv3, and YOLOv4 on the UAV aerial vehicle dataset but without considering the impact of vehicle occlusion, shooting angle, and lighting conditions on the model. Zhang et al. [32] propose a novel multi-scale adversarial network for improved vehicle detection in UAV imagery. The model performs great in images from different perspectives, heights, and imaging situations. However, the classification of vehicles is not specific enough, with only two categories: large vehicles and small vehicles.

Because of its excellent detection accuracy and quick inference, YOLOv5 [33] is applied extensively in various fields for practical applications. Niu et al. [34] used the ZrroDCE low-light enhancement algorithm to optimize the dataset and combined it with YOLOv5 and AlexNet for traffic light detection. Sun et al. [35] employed YOLOv5 to identify the marks added to bolts and nuts, from which the relative rotation angle was calculated to determine whether the bolts were loose. Yan et al. [36] applied an enhanced model based on YOLOv5 to apple detection, which improved the detection speed and reduced the false detection rate of obscured targets.

To reduce the false and missed detection rates of vehicle detection tasks, this paper conducts research to refine YOLOv5s, the smallest network in YOLOv5 version 6.1. The details are outlined as follows:

(1) In this paper, a smaller detection layer is added to the three detection layers of the original network. It makes the network more sensitive to small targets in high-resolution pictures and strengthens the multi-scale detection capability of the network.
(2) We introduce the Bifpn structure [37] based on YOLOv5, which strengthens the feature extraction and fusion process. Bifpn enables the model to utilize the deep and shallow feature information more effectively and thus obtain more details about the small and occluded objects.
(3) YOLOv5s adopts the NMS algorithm, which directly deletes the one with low confidence in two candidate frames that overlap too much, resulting in missed detection. Therefore, we use the Soft-NMS (soft-non-maximum suppression) algorithm [38] to optimize the anchor frame confidence, effectively alleviating the missed detection caused by vehicle occlusion.

## 2. Related Work

### 2.1. Overview of YOLOv5

**Figure 1:**
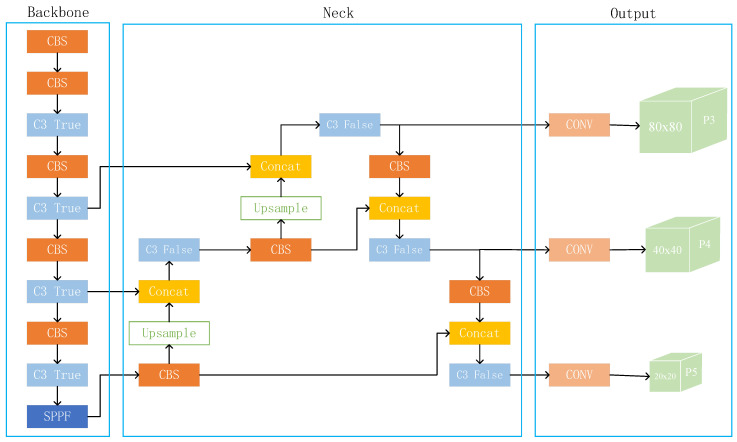
The framework of the YOLOv5s algorithm.

YOLOv5 is a single-stage target detection algorithm released by Ultralytics in 2020 that consists of four structures: YOLOv5s, YOLOv51, YOLOv5m, and YOLOv5x. The model works by dividing the image into multiple grids, and if the center of the target falls within a grid, that grid is responsible for predicting the object. YOLOv5s is the most miniature model in depth and width among these four models. With the increase in model size, although the detection accuracy improves, the detection speed also becomes slower. As shown in Figure 1, YOLOv5s network is mainly categorized into four parts: input layer(input), backbone, neck, and prediction layer (head).

**Figure 2:**
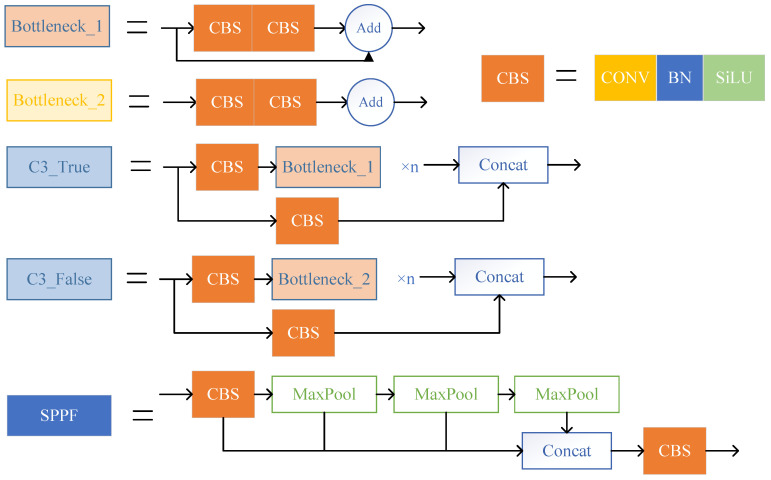
Structure diagram of CBS, C3, and SPPF modules.

The primary function of the input layer is to unify the size of the input image into a fixed size. The backbone section, which includes the CBS, C3, and SPPF modules, is primarily responsible for extracting essential information from the input picture. The structure of each module is shown in Figure 2. The Neck portion of YOLOv5 employs a mixed structure of FPN [39] and PAN [40]. FPN transfers semantic information from deep to shallow feature maps, while PAN conveys localization information from shallow to deep feature layers. The combination of the two may aggregate characteristics from multiple backbone levels to different detection layers, enhancing the feature fusion capacity of the network.

The YOLOv5 target detection algorithm, which is still being updated and iterated, has reached high accuracy and speed by absorbing the benefits of other detection methods. Additionally, it is easy to implement. As a result, in this paper, we selected YOLOv5s as the baseline and used a series of experiments to develop a model more appropriate for aerial vehicle identification.

### 2.2. Adding a Prediction Layer for Tiny Objects

**Figure 3:**
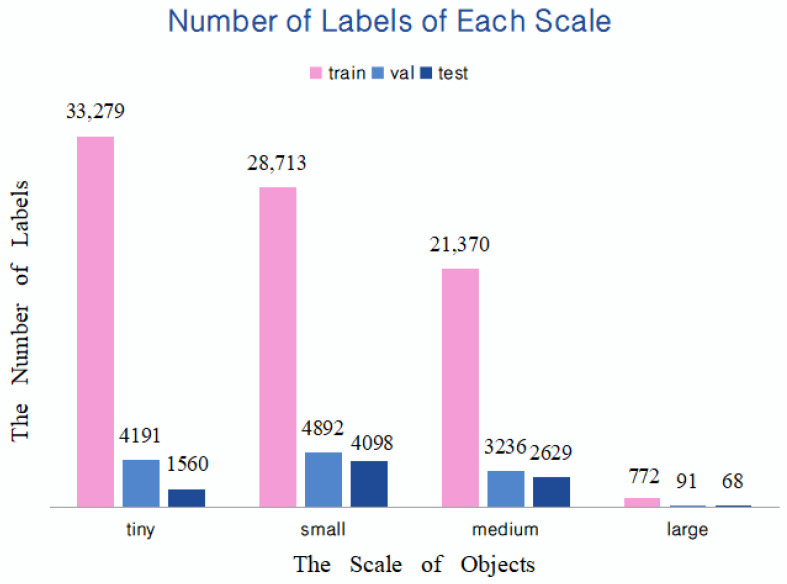
Number of objects at different scales in this dataset.

The maximum downsampling step of the YOLOv5s network is 32. Therefore, a resolution less than 32 × 32 pixels is regarded as a small target [41], greater than 96 × 96 pixels is defined as a large target, and in between is classified as a medium target. Since there are a large number of targets with tiny scales in the pictures taken by UAVs, we further subdivide the targets with a resolution less than 32 × 32 pixels into two cases of tiny (resolution < 16 × l6 pixels) and small (16 × 16 pixels < resolution < 32 × 32 pixels). The obtained target scale distribution is shown in Figure 3. It can be found that the number of tiny objects in train, val, and test are all significant. Therefore, it is essential to customize a detection layer more suitable for detecting tiny targets.

The YOLOv5s network has three detection layers, P3, P4, and P5, with feature map sizes of 80 × 80, 40 × 40, and 20 × 20, respectively. The larger size of the feature map is responsible for detecting smaller objects. The largest 80 × 80 feature map corresponds to an input size of 640 × 640, and the receptive field size of a grid in the feature map is 8 × 8. If the height or width of a tiny vehicle in the original image is less than 8 pixels, it is difficult for the network to learn the features of the object. The new P2 detection branch can detect targets at the 4 × 4 pixel level while configuring anchor boxes of smaller size, thus effectively reducing the missed detection of tiny vehicles.

**Figure 4:**
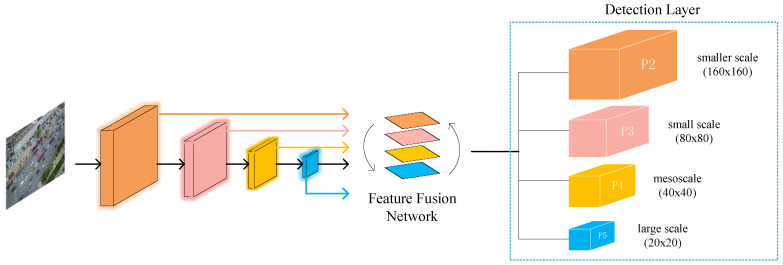
YOLOv5s algorithm framework with the added tiny object detection layer.

From Figure 4, we can see that the first C3 module in the Backbone outputs a feature map of 160 × 160 after two downsamplings, while the second C3 module produces a size of 80 × 80. We fuse the feature map of 160 × 160 with the output of the second C3 module after upsampling to obtain the detection branch P2. In this way, the input of P2 derives mainly from the shallow convolutional layer and contains more information related to shape, position, and size. This information facilitates the model to discriminate fine-grained features more accurately, thus improving the capability to detect small targets.

### 2.3. Enhancing Feature Fusion with Bifpn

The Neck part of YOLOv5 uses a combined FPN and PAN structure. When it comes to feature extraction, the shallow network has a higher resolution and more precise position information. On the other hand, the deeper network has a more extensive receptive field and more high-dimensional semantic information that aids with object categorization. FPN facilitates semantic information from deep feature maps to shallow feature maps, while PAN accomplishes a high degree of integration between shallow localization information and deep semantic information. The combination of FPN and PAN, which aggregates parameters from multiple backbone levels to distinct detection layers, significantly improves the feature fusion capabilities of the network.

**Figure 5:**
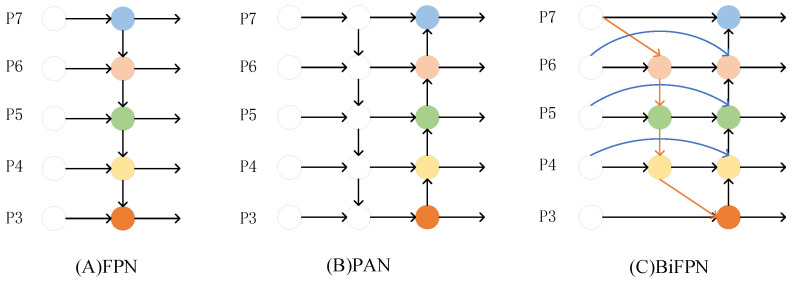
Schematic diagram of different feature fusion structures.

Nevertheless, there is a problem in that input to the PAN structure is largely feature information processed by FPN, with no original feature information taken from the backbone network. This issue may cause the optimization direction of the model to be biased, affecting the detection impact. The BiFPN first simplifies the PAN structure by removing the nodes with only one input and output edge. Then, an extra edge is added between two nodes at the same level to fuse more differentiated features, and the structure is shown in Figure 5C. The original BiFPN would assign various weights to different input features according to their importance, and this structure is utilized frequently to encourage feature fusion. The introduction of BiFPN with weights, however, increases the number of parameters and calculations for the dataset in this research, and the detection effect is not satisfactory.

**Figure 6:**
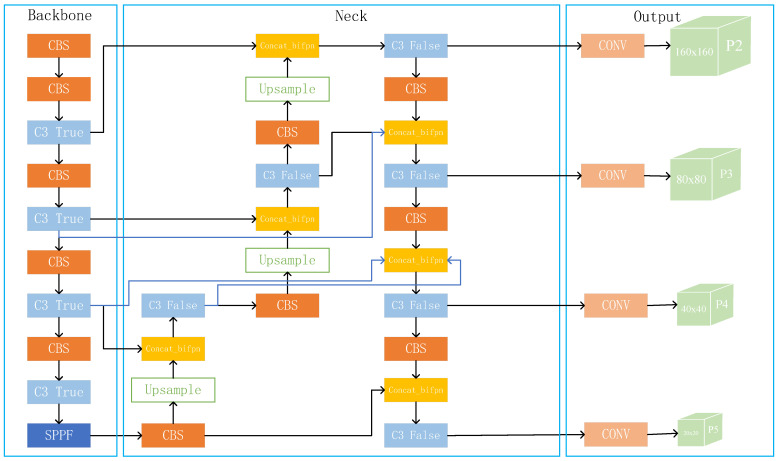
Network structure diagram after improving Neck and Head parts.

Because the motivation for introducing BiFPN is that PAN can obtain more original feature information as input, we remove the weighted part and only reference its cross-scale connection way. By introducing the de-weighted BiFPN, the trade-off between accuracy and efficiency is considered, making the feature fusion process more reasonable and efficient. In this way, each node of PAN has one input edge from the backbone network, making the training process have continuous involvement of the original features and preventing the model from deviating from the expectation during the training process. The feature information of tiny targets is already relatively insufficient, and the features are easily missing after several convolutions. As shown in Figure 6, part of the input of the added prediction layer is from the first C3 module. This module retains most of the original feature information. Thus, more features about the tiny objects can be obtained, and the detection performance of the model can be improved.

### 2.4. Introducing Soft-NMS to Decrease Missed Detections

Instead of the NMS algorithm adopted by YOLOv5, Soft-NMS is used in this paper. The NMS algorithm selects the one with the highest confidence among all the predictor frames then conducts IoU operations sequentially with other predictor frames. For a prediction box whose IoU value exceeds the set threshold, it is directly deleted. During peak commuting hours, the vehicle density in the images captured by the UAV is high and closely aligned. In this circumstance, using the NMS algorithm suppresses many anchor frames that initially belonged to different targets, resulting in the missed detection of obscured vehicles.The NMS algorithm is shown in Equation (1).

(1) $$s_{i}=\left\{\begin{array}{cc} s_{i}, & \mathrm{IOU}\left(M,b_{i}\right)<N_{t}\\ 0, & \mathrm{IOU}\left(M,b_{i}\right)⩾N_{t} \end{array}\right.$$

where $b_{i}$ and $s_{i}$ denote the ith predictor box and its score, respectively, and $N_{t}$ is the set threshold. *M* indicates the candidate box with the highest score. When the IoU of *M* and $b_{i}$ is greater than the threshold, the score $s_{i}$ of b is directly set to 0, likely to erroneously remove some prediction boxes containing vehicles.

Unlike the NMS method, Soft-NMS selects M as the benchmark box then calculates the IoU between M and the neighboring predictor boxes. This adjacent prediction frame is not suppressed when the IoU value is less than the set threshold. When the IoU value is greater than the set threshold, the penalty function attenuates the scores of the prediction frames that overlap with the reference frame instead of directly setting the scores to 0. By penalizing the scores of prediction frames with large IoU values, anchor frames with larger overlap areas get higher penalty coefficients and more minor scores $s_{i}$. Thus, there is a chance they are preserved during the suppression iterations, avoiding the situation where highly overlapping prediction frames contain targets but are removed.

The expression of the Soft-NMS algorithm is given in Equation (2).

(2) $$s_{i}=\left\{\begin{array}{cc} s_{i}, & \mathrm{IoU}\left(M,b_{i}\right)<N_{t}\\ s_{i}e^{-\frac{\mathrm{IoU}{\left(M,b_{i}\right)}^{2}}{\sigma }} & \mathrm{IoU}\left(M,b_{i}\right)⩾N_{t} \end{array}\right.$$

where σ is the hyperparameter of the penalty function. Combining with Equation (2), it can be seen that when the overlap of two boxes is higher, the value of $\mathrm{IoU}{\left(M,b_{i}\right)}^{2}$ is larger and $s_{i}$ is smaller. So the predicted box obtains a smaller score but can be retained instead of directly deleted, thus avoiding the missed detection of overlapping vehicles.

**Figure 7:**
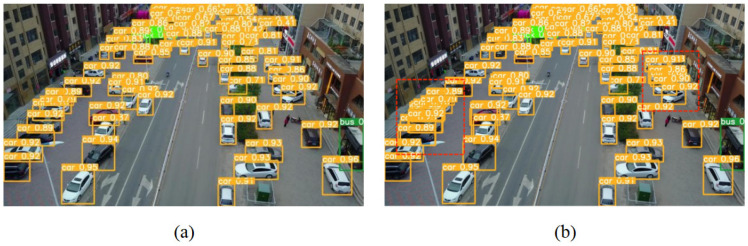
Comparison of YOLOv5s algorithm detection results before and after using Soft-NMS. (**a**) The detection performance of YOLOv5; (**b**) The detection performance of YOLOv5 after the introduction of Soft-NMS.

Figure 7a compares the detection performance of YOLOv5 using NMS and Soft-NMS as prediction frame screening algorithms. By focusing on the red dashed box in Figure 7b, it can be found that the application of the Soft-NMS algorithm successfully decreases the number of missed vehicles in the densely arranged region and enhances the detection performance of the model in the high-overlap scenario.

## 3. Experiments

### 3.1. Experimental Setup

In our experiments, the operating system was Linux, the CPU was an Intel(R) Xeon(R) Platinum 8358P CPU @ 2.60 GHz, the GPU was an RTX A5000-24 GB, and the framework was Pytorch. The experimental settings were based on the official YOLOv5 default parameters, including an adaptive anchor strategy and mosaic data enhancement. The parameters of the training process are set as shown in Table 1.

### 3.2. Dataset Description

**Figure 8:**
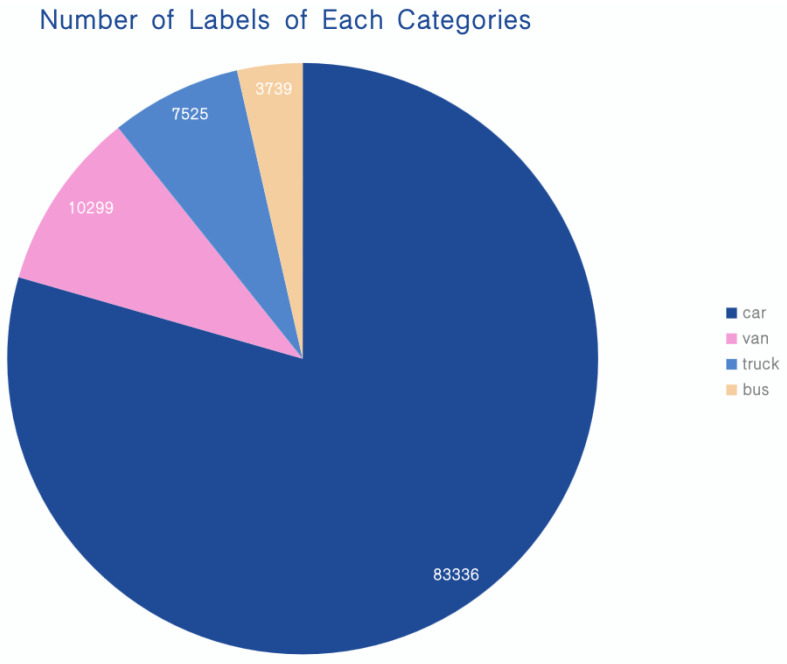
Pie chart describing the proportion of instances of labels for each category.

Vehicles of four categories—car, van, truck, and bus—were selected for training, validation, and testing by collating the open-source dataset VisDrone2019-DET [42]. The number of labels for each category is shown in Figure 8.

**Figure 9:**
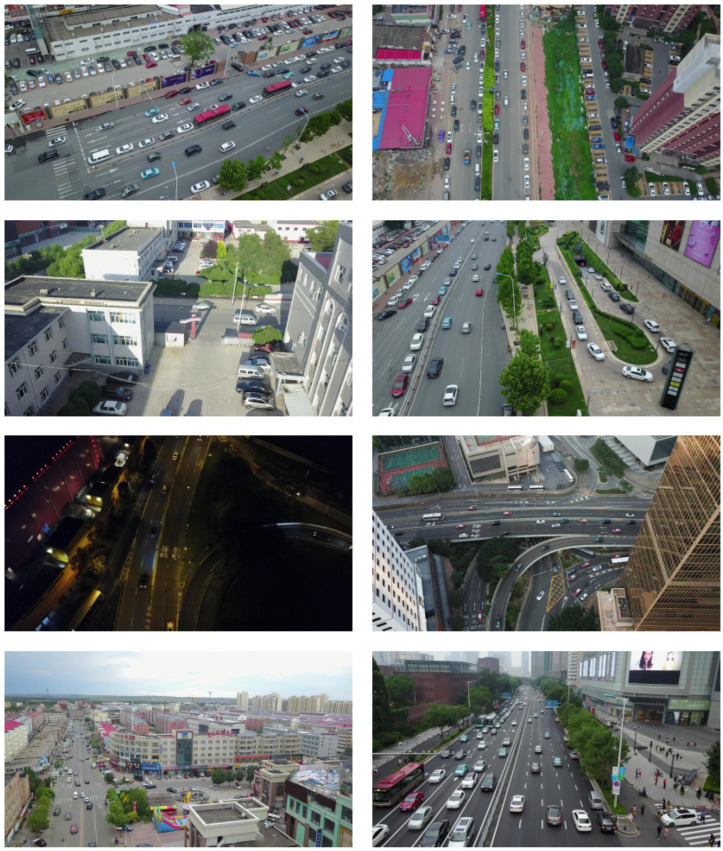
A few examples of the images in the dataset used in this article.

There are ten categories in the VisDrone2019-DET dataset labels, several of which have few vehicle targets in the photos. As a result, we carefully selected 3650 photos from the original dataset as the experimental dataset for this paper to increase the training efficiency of the model. Figure 9 shows some of the images in the dataset of this paper. The dataset is usually divided into a training set, a validation set, and a test set. Among them, the training set is responsible for training the model, the validation set is used to optimize the parameters continuously, and the test set is assigned to evaluate the model. If the data distributions of these three sets differ greatly, this may affect the generalization ability of the model in real scenarios. Therefore, it is essential to allocate the dataset randomly, and the common ratios are 8:1:1 and 7:2:1. In this work, we randomly partition the dataset roughly according to the proportion of 8:1:1 to obtain 2800 in the training set, 350 in the validation set, and 500 in the test set.

### 3.3. Data Pre-Processing

**Figure 10:**
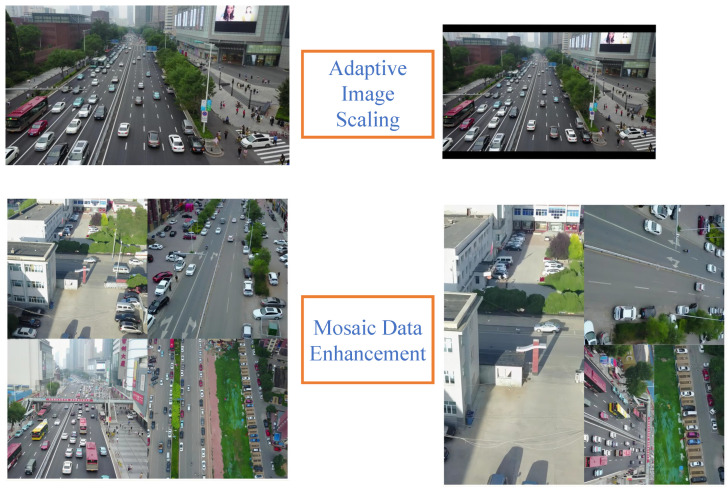
Effect of adaptive image scaling and mosaic data enhancement.

We applied adaptive image scaling and mosaic data enhancement to pre-process the dataset. Because many original images have different aspect ratios, they need to be scaled and padded before feeding them into the model. If the sides are filled with more black borders, it will lead to redundant information and affect the training speed. Therefore, we use adaptive image scaling, which can adaptively add the least amount of black borders to the original images, thus speeding up the learning speed of the model. Mosaic data enhancement selects four original randomly scaled images that are cropped and arranged, and then it stitches them into a new image. This data enhancement method can effectively boost the ability to detect small targets. Figure 10 shows the two data preprocessing types.

### 3.4. Evaluation Metrics

In this study, AP and mAP are used as the evaluation metrics of the model. The average precision considers the precision (*P*) and recall (*R*) of the model. FLOPs, parameters, and FPS are applied to estimate the model size. The equations for precision P, recall, and mAP are as follows.

(3) $$P=\frac{TP}{TP+FP}$$

(4) $$R=\frac{TP}{TP+FN}$$

(5) $$AP=\int_{0}^{1}P\left(R\right)dR$$

(6) $$mAP=\frac{\sum_{i=1}^{N}AP_{i}}{N}$$

The terms TP, FP, and FN indicate the number of objects that were correctly detected, wrongly detected, and undiscovered, respectively. P is the precision, indicating how many vehicles predicted to be in a certain category actually belong to that category in our manuscript. R is the recall, which shows the proportion of vehicles in a category in the dataset that are correctly predicted. It is easy to see that precision focuses on the accuracy of the detected vehicle category, while recall pursues the detection of more vehicles in a particular type. AP is the area under the P-R curve for a single class. Finally, mAP indicates the average AP of all categories and is a composite measure of detection performance.

## 4. Results

### 4.1. Ablation Experiment

Three structures are explored to improve the YOLOv5s algorithm in this paper. The first is the addition of a new detection layer, P2, to enhance the recognition capability of small target vehicles. The second is the introduction of a de-weighted BiFPN to make the feature fusion process more reasonable and practical. The third is the use of Soft-NMS as a prediction frame filtering algorithm to improve the detection performance of overlapping and occluded vehicles. We designed the corresponding ablation experiments to verify the effectiveness of YOLOv5 after adding different modules, and the results are shown in Table 2. The number of parameters and computation of the model modestly increased compared with the baseline model after adding the P2 detection layer, as seen from the data analysis in the table. By further introducing BiFPN, however, the number of parameters and calculations are reduced significantly while the accuracy is guaranteed. The three improvement strategies are combined to produce the improved model, YOLOv5-VTO. While the addition of Soft-NMS reduces the AP of “car” compared to using only P2 and Bifpn, the remaining categories of AP are improved. Because the model has achieved excellent detection performance for “car”, we think “van”, “truck”, and “bus” are more in demand for a boost in AP. In addition, the substantial improvement of mAP also indicates that the introduction of Soft-NMS plays a great role in enhancing the comprehensive performance of the model. Of course, it is also clear in Figure 7 that Soft-NMS does decrease the missed detection of closely arranged vehicles.

Compared with the benchmark model, the two comprehensive indexes of mAP@0.5 and mAP@0.5:0.95 are improved by 3.7% and 4.7%, respectively, effectively improving the accuracy of aerial vehicle detection. Although there is a small increase in the number of parameters and computation compared with the benchmark model, it is discovered that the modified model can still satisfy the requirements of real-time detection in the following comparative experiments. The ablation experiments demonstrate that the approach used in this paper is excellent in the UAV aerial vehicle detection task, outperforming the base model in scenarios with tiny targets and more overlapping occluded objects.

**Figure 11:**
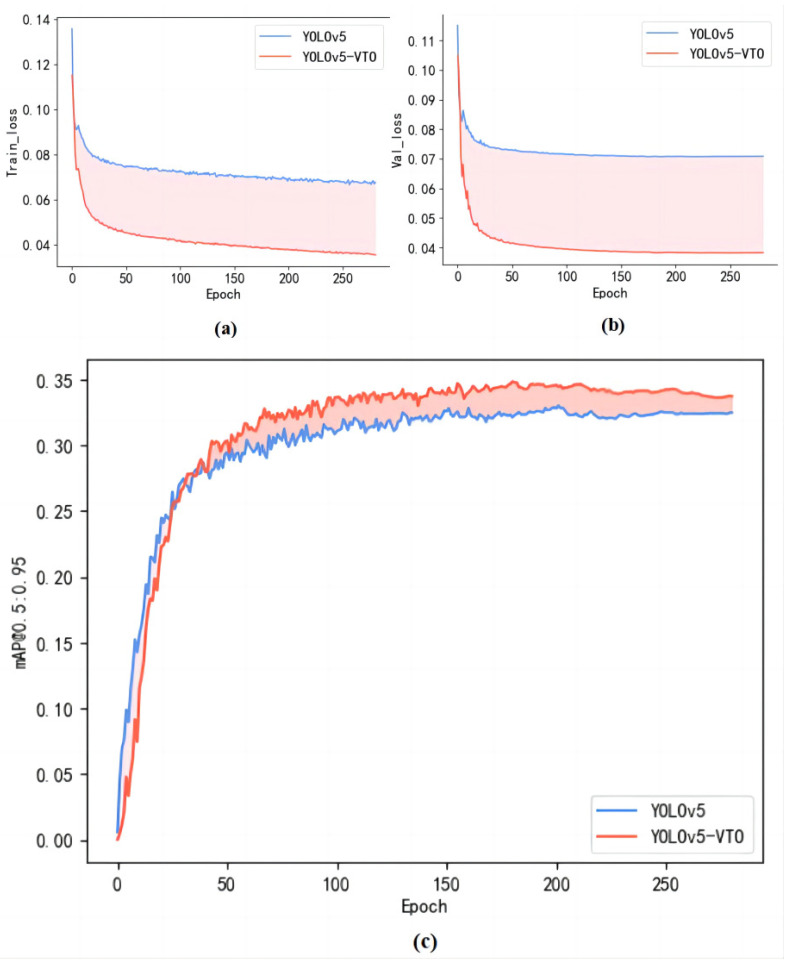
Comparison of the training curve between our model and YOLOv5s. (**a**) The loss function curve of the training set; (**b**) The loss function curve of the validation set; (**c**) The mAP curve.

Throughout the training procedure, the YOLOv5 and the YOLOv5-VTO models in this article use the same dataset and parameter settings. The mAP and loss comparison graphs of the two models are plotted according to the log files saved during the training process, as shown in Figure 11.

Figure 11a shows that the model obtained a higher mAP after improvement. In contrast, Figure 11b,c illustrate no obvious overfitting problem in the training process. Furthermore, compared to the baseline model, the overall loss value of the model in the training and validation sets is much lower.

**Figure 12:**
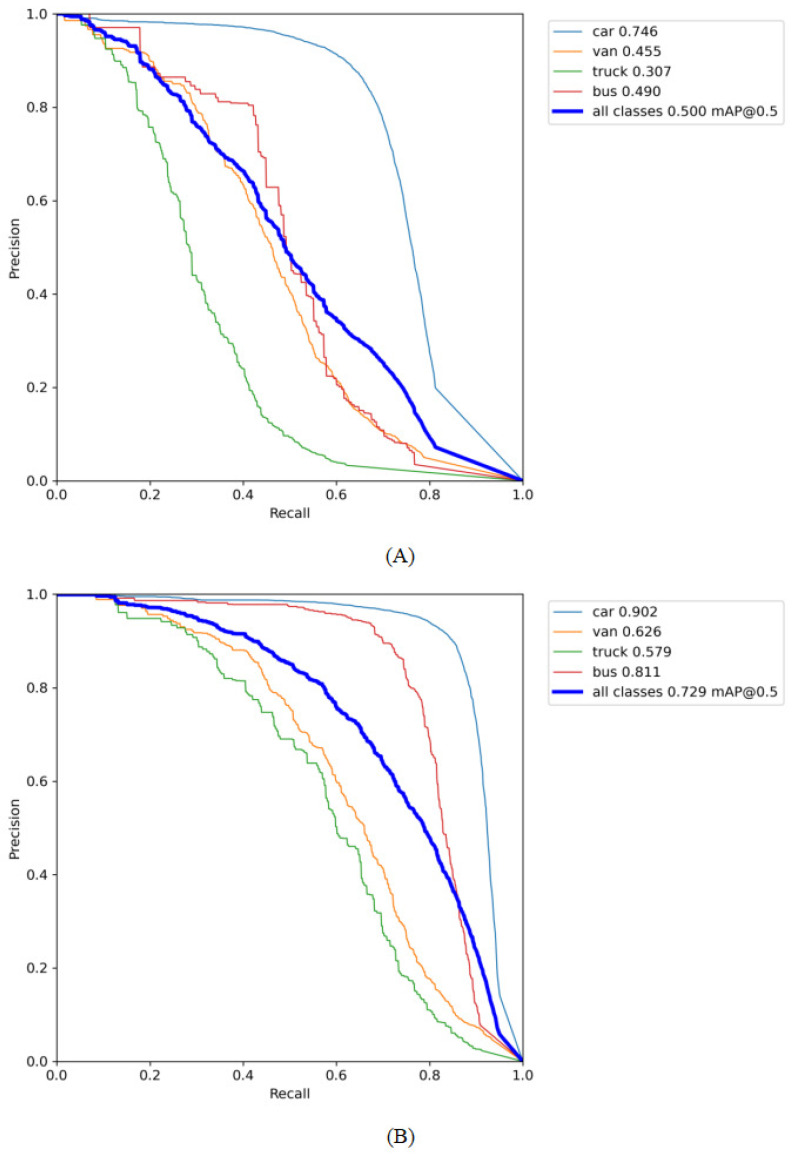
PR curve comparison: (**A**) PR curve of YOLOv5s and (**B**) PR curve of improved YOLOv5s.

We plotted the P-R curves shown in Figure 12 based on the log files regarding precision and recall generated during the training of the model. Because Soft-NMS is a prediction-frame optimization method in the prediction phase, it is not involved in the model training process. Therefore, Figure 12B is only a curve obtained by training the model after adding P2 and Bifpn. The area below the P-R curve indicates the AP of a category, so that the closer the curve is to the upper right corner, the better the overall performance of the algorithm.

It is not difficult to find that precision and recall have an inverse relationship in the P–R curve. This is because when the model pursues a high accuracy rate, it will be more conservative in prediction. Some samples with low confidence cannot be predicted confidently, and recall will be reduced accordingly. The importance of these two metrics is different in various scenarios. Therefore, we have to make a trade-off between precision and recall according to the needs of specific problems.

We can see that the model enhances the detection capability of vehicles, especially in the categories of “truck” and “bus,” by comparing the PR curves before and after the model improvement in Figure 12. However, compared with the “car” category, the performance of the updated model in the “truck” and “van” categories still needs to be improved, and the average accuracy of the best and worst detection categories is 0.902 and 0.579, respectively. The reason for this is that there are fewer target instances in the “truck” and “van” categories in the dataset compared to the “car” category. In addition, “truck” has many types of shapes, resulting in complex and variable feature information, which increases the difficulty of detection. As a result, we will continue looking for approaches to boost the detection effect of the model in the following stage, such as data supplementation and enhancement for the relevant categories.

### 4.2. Comparative Experiment

We compare YOLOv5-VTO with a series of target detection algorithms, including YOLOv5s, Faster-RCNN, SSD, YOLOv3-tiny, YOLOv7-tiny [43], and Efficientdet-D0, to further evaluate the advantages of the algorithms in this study for the vehicle identification task. All models involved in the comparison were trained and validated using the same dataset, and the experimental data are presented in Table 3.

The experimental results of different algorithms in Table 3 show that the YOLOv5-VTO algorithm proposed in this paper achieves the highest mAP compared to other mainstream detection models. Compared with the benchmark YOLOv5s, the proposed model has significantly improved mAP@0.5, mAP@0.5:0.95, Precision, and Recall while keeping the detection speed pretty much the same.

As a representative of the anchor-free detection model, the Efficientdet algorithm has room for improvement in mAP. On the other hand, the two-stage detection algorithm Faster-RCNN is slower owing to the need to extract feature vectors from feature regions during the testing phase. A high accuracy rate is achieved for the same single-stage detection algorithm SSD. However, the lack of a low-level feature convolution layer of SSD leads to inadequate features extracted from small target vehicles, resulting in a high number of missed detections and low recall. Given the need for the model to detect in real-time, two miniature volume versions, YOLOv3-tiny and YOLOv7-tiny, are selected for comparison in this study. YOLOv7-tiny achieved good results in terms of recall and FPS based on the experimental data. Yet the model provided in this paper still has benefits in several other metrics, such as mAP, especially mAP@0.5:0.95. YOLOv3-tiny has a significant gap compared to YOLOv5-VTO in various indexes except for FPS. Although our model is lower than these two algorithms in terms of detection speed, it may still satisfy the demand for real-time detection.

In summary, the overall performance of this model is remarkable compared with other models, and the balance of detection accuracy and detection speed is achieved, which verifies the effectiveness of this model.

### 4.3. Visualizing the Detection Performance of Different Models

**Figure 13:**
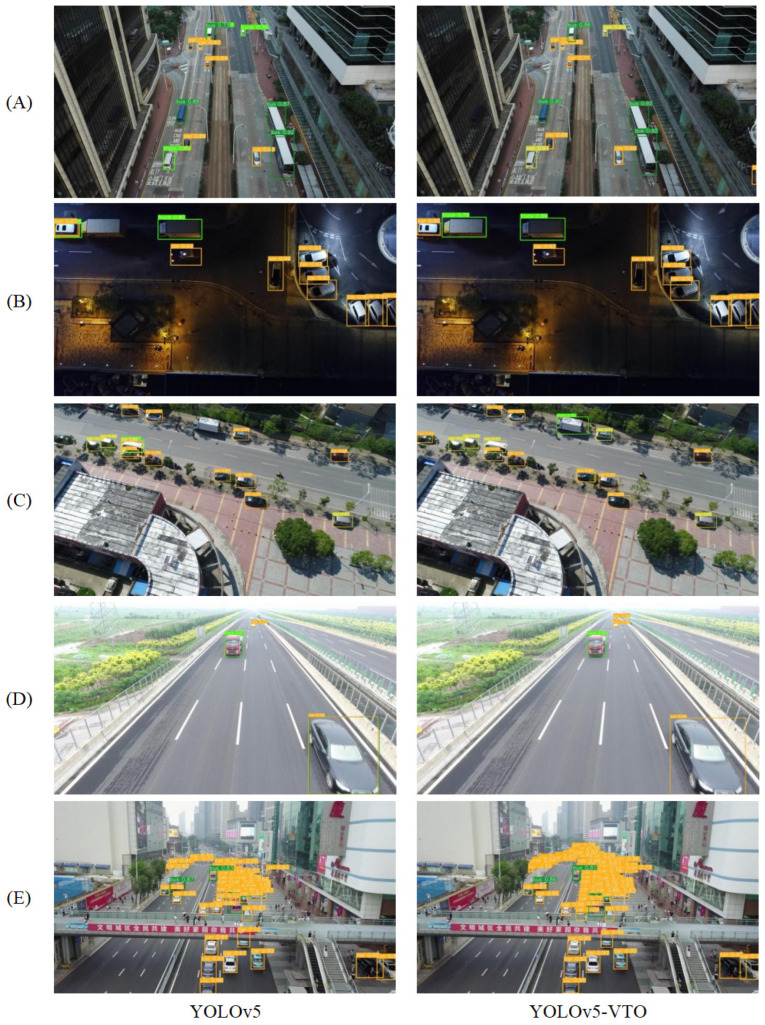
Comparison of YOLOv5s algorithm detection results before and after improvement. (**A**) Improved model reduces false detections; (**B**) Mitigates missed detections in low-light scenes; (**C**) Improved model reduces missed detections; (**D**) Improved model enhances the detection performance for small targets; (**E**) Improved model reduces missed detection of mutually obscuring vehicles.

We have demonstrated the comparison of the detection effect before and after the modification, as shown in Figure 13, to evaluate the model more intuitively. Figure 13A shows that the model YOLOv5-VTO can reduce the false detection of vehicles. It can be observed from comparing the findings of groups B and C that the revised model decreases the rate of missed detection and remains effective even in scenes with insufficient light. From the contrast results of both groups D and E, it can be found that the detection performance of the model in this paper for tiny targets is improved. The above visualization results show that our model achieves better detection performance for tiny and obscured vehicles in aerial images.

## 5. Conclusions and Future Works

We propose an enhanced model, YOLOv5-VTO, based on YOLOv5s to improve the detection performance of obscured vehicles and tiny vehicles in aerial images. Above all, a new detection branch, P2, that can discover tiny targets accurately is added to three detection layers of the baseline model. Then, the bi-directional feature pyramid (BiFPN), rather than the PAN structure of the original model, is adopted to achieve the excellent fusion of feature information of multiple scales to reduce the conflict between the fusion of features of different scales. By visualizing the detection results at last, we find that the Soft-NMS algorithm plays a good role in the scenario where vehicles block each other.

The experimental results indicate that the improved algorithm is more effective than the original YOLOv5s algorithm. Further, the detection speed can still achieve 30FPS, which can meet the demands of real-time detection. Although soft-NMS can improve the detection of obscured vehicles, it also slightly reduces the AP of some categories, such as “car”. Therefore, our following research will focus on how to mitigate this side effect after introducing Soft-NMS. Furthermore, there are still many uncertainties that limit the detection speed of the model, such as the large number of vehicles in the image, changing lighting conditions, the selection of the anchor box, the setting of the confidence threshold, and the deployment of high-performance hardware devices. Therefore, in future work, we will explore how to satisfy real-time detection applications under constraints. 

## Figures and Tables

**Table 1:** Parameters of training.

Parameters	Configuration
Image size	640 × 640
Learning rate	0.01
Momentum	0.937
Data enhancement	MOSAIC
Total epoch	300
BatchSize (training)	32
BatchSize (testing)	1
Network optimizer	SGD

**Table 2:** The comparison of the performance with different modules.

P2	Bifpn	Soft-NMS	AP				mAP@0.5	mAP@0.5:0.95	Params(m)	GFLOPS
			Car	Van	Truck	Bus				
			0.890	0.578	0.521	0.783	0.693	0.470	7.03	15.8
✓			0.901	0.631	0.564	0.820	0.729	0.490	7.69	27.0
✓	✓		0.902	0.626	0.579	0.811	0.729	0.488	7.38	20.0
✓	✓	✓	0.872	0.630	0.598	0.819	0.730	0.517	7.38	20.0

**Table 3:** Comparison of detection performance of different algorithms.

Model	mAP@0.5	mAP@0.5:0.95	Precision	Recall	FPS
Faster R-CNN	0.713	0.400	0.665	0.556	20.4
SSD	0.650	0.450	0.801	0.505	30.9
YOLOv3-tiny	0.546	0.287	0.593	0.548	80.5
YOLOv7-tiny	0.721	0.475	0.778	0.651	71.4
Efficientdet-D0	0.665	0.435	0.792	0.620	41.2
YOLOv5s	0.693	0.470	0.762	0.631	37.4
YOLOv5-VTO	0.730	0.517	0.779	0.642	37.0

## Data Availability

The data used in this paper were derived from the following sources available in the public domain [42]: VisDrone-DET2019: The Vision Meets Drone Object Detection in Image Challenge Results.

## Abbreviations

The following abbreviations are used in this paper:

YOLO	You Only Look Once
IoU	Intersection over Union
HOG	Histogram of Oriented Gradients
SIFT	Scale Invariant Feature Transform
FPN	Feature Pyramid Network
PANet	Path Aggregation Network
UAV	Unmanned Aerial Vehicle
NMS	Non-Maximum Suppression
AP	Average Precision
mAP	Mean Average Precision
SVM	Support Vector Machine
SSD	Single Shot Detector
FPS	Frames Per Second
FLOPS	Floating Point of Operations
CBS	Conv BN SiLU
TP	True Positives
FP	False Positives
FN	False Negatives
